# Post-operative Incomplete Quadriplegia Caused by Conversion Disorder in a Female After Gynecological Surgery: A Case Report

**DOI:** 10.7759/cureus.30988

**Published:** 2022-11-01

**Authors:** Maria L McCrackin, Nicole A Weiss

**Affiliations:** 1 Anesthesiology, University of California Davis, Sacramento, USA

**Keywords:** functional neurological disorder, non-organic quadraplegia, non-organic paralysis, post-operative conversion disorder, post-operative quadraplegia, conversion disorder

## Abstract

Conversion disorder (CD) is a psychological disease that presents as neurologic symptoms. This diagnosis of exclusion can include symptoms such as paralysis, blindness, non-epileptic seizures, and movement disorders. We describe a case of a healthy 39-year-old woman who underwent a laparoscopic hysterectomy with a routine general anesthetic and developed incomplete quadriplegia and urinary incontinence post-operatively. Her labs and imaging were all within normal limits and neurology felt her presentation was inconsistent with most organic neurological insults. One month later, she received a diagnosis of conversion disorder.

## Introduction

Conversion disorder (CD), also known as a functional neurological disorder, is associated with psychological stressors and consists of genuine neurologic symptoms without an organic cause or explanation [1]. The estimated occurrence is 50 per 100,000 in the general population [2]. It is at least twice as common in females and occurs in children and adults [1,2]. Published diagnoses of CD in the perioperative setting are limited to several case reports that describe symptoms including non-epileptic seizures on induction, hemiplegia, and quadriplegia after regional and general anesthetics [3,4,5,6]. Here we describe a case of conversion disorder that presented as new onset incomplete quadriplegia in a healthy female after a routine anesthetic. Written authorization was obtained from the patient prior to submission of this case report. This manuscript was written in accordance with the requirements of Health Insurance Portability and Accountability Act of 1996 (HIPAA) privacy regulations.

## Case presentation

A 39-year-old, 70 kg Caucasian female with a past medical history of asthma, migraines, and anxiety, underwent an elective total robotic hysterectomy and bilateral salpingectomy for uterine fibroids. She had no previous anesthetic history. She was appropriately positioned in dorsal lithotomy with Allen® Yellofin® stirrups during the procedure. Her intraoperative course was unremarkable, with a total duration of 301 minutes under general anesthesia. She received standard doses of midazolam, fentanyl, propofol, and rocuronium. Her neuromuscular blockade was reversed with 2 mg/kg of Sugammadex after 4/4 documented twitches. She was extubated and taken to the post-anesthesia care unit (PACU) without issue.

After becoming more alert in the PACU, the patient reported weakness in all her extremities. She did not have numbness, tingling, or foot drop concerning for compression injury. She also went on to develop urinary incontinence. Her review of systems (ROS) was otherwise unremarkable. She denied a personal or family history of neuromuscular conditions (e.g. muscular dystrophy, myasthenia gravis, Lambert-Eaton syndrome) or thyroid problems. Her exam demonstrated normal work of breathing, vital signs within normal limits, and good tone and range of motion in her joints with passive movement. 

Despite low concern for a residual neuromuscular blockade, she was given an additional 400 mg of Sugammadex without improvement in her symptoms. A complete blood count, comprehensive metabolic panel, and thyroid stimulating hormone level were within normal limits. The patient was introduced to the possibility of CD as an explanation for her quadriplegic-like symptoms.

Neurology was consulted for further examination, which was significant for sensory splitting on the scalp, face, chest, arms, and legs on light touch and vibration. The patient was able to wiggle her fingers and supinate her forearms in the plane of the bed, but was unable to volitionally lift her arms off the bed. Her arms were passively brought above her head and she was inconsistently able to hold them in position for a prolonged period of time. She demonstrated a positive drop test (arm fell to the side to miss her face when dropped from above her head) [7]. On the lower extremity exam, she demonstrated antigravity at the hip and sustained knee flexion with encouragement. She was able to wiggle her toes. Her reflexes (biceps, brachioradialis, triceps, patellar, Achilles) were all 2+ bilaterally. Due to the discrepancy between the patient’s symptoms and exam with patterns seen in genuine neurological diseases, neurology did not recommend initiating a stroke code or in-house imaging.

Approximately six hours later, the patient had complete recovery of her bilateral arm weakness but continued to have weakness in her lower extremities. Physical therapy evaluated the patient and was able to observe her sit and stand with minimal support for prolonged periods of time. She was noted to have unique gait mechanics (i.e. able to advance bilateral extremities via sliding on the floor versus a true swing phase), but was deemed safe for discharge home with her spouse and a front-wheeled walker without further inpatient consults. Of note, she was discharged with an indwelling urinary catheter as she was unable to void.

The patient was referred for appropriate postoperative follow-up, physical therapy, neurology, and psychiatry upon discharge from the hospital. Given persistent bilateral lower extremity weakness, magnetic resonance imaging (MRI) of the spine was obtained which showed one focal left lateral disc protrusion abutting upon the exit point of the left L3 nerve in the L3-4 foramen. Electromyography (EMG) of all extremities was within normal limits. Computed tomography (CT) scan of the abdomen and pelvis was performed given persistent urinary symptoms, which ruled-out complications from her hysterectomy. She received a thorough and formal evaluation with psychiatry, including identification of life stressors, past trauma, and coping mechanisms. One month after her surgery, she officially received the diagnoses of trauma and stressor-related disorder, and conversion disorder.

## Discussion

Diagnosing new onset neurological symptoms as CD can be quite challenging for several reasons. Perhaps most notable is the fear of a physician missing a potentially devastating neurological disease, despite CD having a misdiagnosis rate of only 4% [8]. It can also be difficult for providers to determine which diagnostic tests to perform and when to stop a diagnostic workup. Adequate time must be spent on psychological evaluation to determine the presence and contribution of life stressors to a diagnosis of CD. Lastly, and likely most challenging, is the ability of physicians to distinguish between a patient exhibiting genuine symptoms versus feigning symptoms for some personal benefit (such as with malingering, Munchausen’s, etc). There have been several editorials published on the importance of utilizing a multidisciplinary approach to the diagnosis of CD for these reasons--specifying the benefits of neurological expertise in identifying the non-anatomical pattern of a patient’s symptoms and psychiatric expertise in both identifying psychological risk factors and heading treatment [9,10]. There has also been a shift in acceptance of somatic symptom disorders as important diagnoses to keep on the differential. For suspected psychological conditions in patients with physical complaints, speaking matter-of-factly to the patient can aid in diagnosis acceptance and treatment [11].

## Conclusions

Patients with CD exhibit genuine neurologic symptoms despite a negative neurological workup. For our patient who emerged from anesthesia with new-onset weakness in all four extremities, we had to rule out more out obvious causes related to her surgery and anesthetic--including residual neuromuscular blockade, nerve compression injury secondary to positioning, metabolic or electrolyte derangements, stroke, or infection. We relied on our physical exam, ROS, and the expertise of our neurology colleagues to differentiate true paresis from non-organic paresis. Neurology also assisted in determining the need for any emergent imaging, which was deferred while the patient was in the PACU.

The official diagnosis of CD came after appropriate outpatient follow-up with neurology and psychiatry. At the time of this publication, our patient unfortunately still suffers from lower extremity weakness and is still receiving treatment for CD. 
